# Exploring needs and requirements for a prototype device measuring physical activity in pediatric physical therapy: A qualitative study

**DOI:** 10.1371/journal.pone.0305968

**Published:** 2024-06-25

**Authors:** Barbara Engels, Corelien J. J. Kloek, Marleen E. Sol, Eline A. M. Bolster, Elles M. W. Kotte, Harriët Wittink, Raoul H. H. Engelbert, Jan Willem Gorter, Manon A. T. Bloemen

**Affiliations:** 1 Research Centre Healthy and Sustainable Living, Research Group Lifestyle and Health, Utrecht University of Applied Sciences, Utrecht, The Netherlands; 2 UMC Utrecht Brain Center and Center of Excellence for Rehabilitation Medicine, Utrecht University, Utrecht, the Netherlands; 3 Research Centre Healthy and Sustainable Living, Research Group Innovation of Human Movement Care, Utrecht University of Applied Sciences, Utrecht, The Netherlands; 4 Fitkids Foundation, Amsterdam, The Netherlands; 5 Centre of Expertise Urban Vitality, Faculty of Health, Amsterdam University of Applied Sciences, Amsterdam, The Netherlands; 6 Department of Rehabilitation Medicine, Amsterdam UMC Location University of Amsterdam, Amsterdam, The Netherlands; 7 Department of Rehabilitation, Physical Therapy Science and Sports, UMC Utrecht Brain Center, University Medical Center Utrecht, Utrecht, The Netherlands; 8 CanChild, Department of Pediatrics, McMaster University, Hamilton, Ontario, Canada; Universidad Santiago de Cali, COLOMBIA

## Abstract

**Aims:**

To analyze needs and requirements of Pediatric Physical Therapists (PPTs), parents, children and adolescents with and without developmental disabilities in the future use of an activity monitor prototype (AM-p) in everyday clinical practice.

**Methods:**

Qualitative exploratory study with a thematic analysis approach, based on Braun and Clarke’s six steps. Codes derived from the analysis and central themes were collated, based on Fleuren et al.’s groupings of determinants.

**Results:**

We interviewed 25 PPTs, 12 parents, and 12 children and adolescents. Within four groupings of determinants, we found nine themes: 1) development of information materials; 2) application: output visualization and ease of use; 3) design; 4) relevance and acceptance; 5) shared decision-making; 6) compatibility in daily living; 7) finances, 8) time, and 9) legislation and regulations.

**Conclusions:**

End-users have similar basic needs, with individual fine-tuning to be addressed during further development of the AM-p. A child-friendly design, information material, and an easy-to-use application to read and interpret results, need to be developed. Efficient training for PPTs is important for the use of the AM-p and analysis of results. Communication between PPTs and children as well as parents enhances shared decision-making. We recommend involving diverse end-users to enable maximum customization of the AM-p.

## Introduction

An active lifestyle is important because physical activity (PA) is positively related to children’s present and future physical health, psychosocial development, creativity, and emotional well-being, while mitigating chronic conditions [1–3]. Unfortunately, not even 50% of children and adolescents without and with developmental disabilities, are active enough to profit from those health benefits [3–6]. Children with disabilities are at higher risk of serious health problems related to inactivity than children without disabilities [7]. Pediatric physical therapists (PPTs) are advocates of PA, often supporting children with disabilities and their families to function independently and promote active participation in daily activities [8]. PPTs work in various settings including hospital, rehabilitation clinic, child care center, preschool and school setting [8, 9]. Assessment of PA levels in children by PPTs is essential to guide their actions. PA assessments can display information on the activity patterns of an individual child or adolescent, as well as changes that have developed. Based on this, a tailored intervention program may be indicated and a child may be followed over time and be supported by the PPT to maintain an active lifestyle [2, 3, 7].

PPTs frequently monitor the level of PA in children with self-report instruments, such as questionnaires or activity diaries [10]. Those instruments are a feasible and valuable method to understand the impact of PA on the lived experience. However, they show a substantial amount of variability in over- or underreporting, tend to have a high recall-bias and seem not to correlate with device-measured PA [10–13]. Additionally, there seems to be a lack of valid and reliable questionnaires for children and adolescents with chronic conditions [14]. Compared to self-report instruments, device-based instruments like wearable activity monitors (AMs) appear to have stronger psychometric properties [10]. The market of consumer grade AMs is growing rapidly but literature is inconclusive about the psychometric properties of consumer grade AMs to measure free-living physical behavior (e.g., PA categories or sedentary behavior) consistently in children and adolescents [15–17]. Furthermore, PPTs need to be able to analyze the data which is complicated with consumer-grade wearables such as the Fitbit [18]. PPTs see children with and without assistive movement devices as crutches or wheelchairs, and using a single AM would be favourable. Literature about ambulatory children with and without developmental disabilities using consumer grade AMs is inconclusive when using step count as outcome measurement [18, 19]. For children using a wheelchair in daily living, research including consumer grade AMs as well as research grade AMs is scarce and present solutions wearing multiple AMs seem not feasible [20, 21].

Available research grade AMs are often not developed for the varying population seen by a PPT, e.g. children with and without a developmental disability, very young children, or individuals using movement aids as walkers, crutches or manually driven wheelchairs [19, 21–24]. Additionally, those AMs show large variations in design, body attachment position, comfort when worn, as well as logistical challenges over amount of equipment and finances [11]. Research grade devices are often not user-friendly for PPTs, as they are time-consuming, expensive and require trained experts for data extraction [11]. Those feasibility issues make it difficult for PPTs to choose an AM for the purpose required with the diverse group of patients seen in their daily working environment.

Currently, there are no user-friendly AMs available in PPT practice. Therefore, our team is validating an AM prototype (AM-p) to determine activity categories (stationary, locomotion, cycling) in children with and without developmental disabilities, including children using a manual wheelchair in daily life. It is a single device with an accelerometer and gyroscope (measurements: 7x4x1.5 cm). It contains a battery, and a chip for internal data storage. The AM-p is still in its early developmental stages. It is not yet customized for end-users such as PPTs, children, adolescents, and their parents. Children and parents need to be able to handle (and accept) the device at home. Our ultimate goal is to develop a feasible AM to monitor PA in children and adolescents with and without developmental disabilities in free-living settings (at home, school, kindergarten). Children and parents need to be able to handle the AM-p unsupervised and for several days. Literature indicates that children should wear an AM three to nine days for an accurate assessment of their PA [25–27]. Early childhood is a critical period for growth and development that can shape health and well-being in children, their families and caregivers [6]. Therefore, the needs and requirements of all age groups in children as well as parents of young children are important. We did not find literature examining needs and requirements of potential end-users in early developmental stages of AMs. Therefore, we see the urge to gather input from all potential end-users of the AM-p related to specific needs and requirements for design and comfort, as well as ways of analyzing and using raw data. We believe that this information is essential for future development and eventual implementation of this device in clinical practice.

The aim of our study is to explore needs and requirements of children, adolescents and their parents, as well as PPTs for the AM-p for monitoring PA in everyday PPT clinical practice.

## Methods

### Study design

We report this exploratory qualitative study according to the Standards for Reporting Qualitative Research [28] to enhance transparency. It has a descriptive design with a thematic analysis approach [29]. The exploratory character is appropriate because of the limited amount of research of introducing new AMs in PPT clinical practice. The underlying paradigm is based on social constructivism, where individuals make sense of knowledge based on their individual and social contexts and have the opportunity to introduce their own topics and insights [30].

### Ethics

The Ethical Committee of Research in Health of the University of Applied Sciences Utrecht, the Netherlands, granted the study exempt from the Dutch Medical Research Involving Human Subjects Act (file number 130-000-2020). Prior to participation, we provided written and verbal information about the research to all participants. All participants ≥16 years gave written informed consent before participating. Parents of participating children between 6–16 years of age and children aged ≥12 years provided written informed assent [31]. The research team consisted of researchers and instructed master’s students of the University of Applied Sciences, Utrecht, the Netherlands. The research team had multiple years of experience with qualitative research and senior researchers (>10 years of experience, MB, EB, EK, CK, MS, RE, JWG) trained and supervised the junior researcher and master students (<5 years of experience, BE and students). Researchers who interviewed participants had no personal relationship with the interviewees.

### Participants and sampling

Among end-users, we defined three participant groups: 1) PPTs; 2) parents of children and adolescents with and without developmental disability; and 3) children and adolescents with and without developmental disability. Recruitment and interviews took place between January and May, 2021. We recruited participants in the Netherlands within our professional network of PPTs and through advertisements on social media (LinkedIn, Facebook). We also distributed flyers and written information about the study to recruit participants. We aimed for maximum variation sampling because we assumed that different backgrounds of end-users could influence participants’ needs and requirements in the development of the AM-p [32]. Therefore, potential participants provided information for a screening: PPTs provided information about age, gender, health-care system worked in, and employee status. Parents provided information about age, gender, education level, and specific details about their child. Children provided information about age, gender, medical diagnosis, walking pattern, Functional Mobility Scale, and adherence to the Dutch Guideline of Physical Activity [33].

For inclusion, PPTs had to be working with children, while parents had to have a child, with or without a developmental disability, between 2 and 18 years of age. We included children ≥ 6 years of age for interviewing, given the development of autobiographic recall, cognitive competence, and language capabilities required for interviews [34]. All participants had to be proficient in Dutch language. Parents of children with an intellectual disability were asked whether their child would be able to talk about the AM-p. If so, those children and adolescents were included. By interviewing three different groups of end-users, we ensured data-triangulation to enhance credibility of findings [35]. The sampling strategy and participant characteristics are presented in Tables 1–3.

**Table 1:** Demographic data of interviewed Pediatric physical therapists (PPTs).

Participant number	Age (years)	Gender	PPT Working experience (years)	Health-care setting (care level)	Employee status
1	42	W^a^	13	Primary	Employee
2	34	W	11	Primary	Manager
3	65	M^b^	43	Primary	Manager, PPT
4	45	W	17	Primary	Manager, PPT
5	35	W	10	Primary	Employee
6	46	W	10	Primary	Self-employed
7	39	W	16	Primary	Manager, PPT
8	37	W	11	Primary	Self-employed
9	26	W	2	Primary	Self-employed
10	28	M	2	Primary	Employee
11	59	M	34	Primary	Self-employed
12	55	W	32	Primary	Manager, PPT
13	33	W	7	Primary	Employee
14	36	W	14	Tertiary	Employee
15	42	W	19	Tertiary	Employee
16	40	W	11	Secondary	Employee
17	29	W	7	Secondary	Employee
18	41	M	13	Secondary	Employee
19	33	W	5	Secondary	Employee
20	33	M	10	Secondary	Employee
21	30	W	5	Secondary	Employee
22	35	W	8	Tertiary	Employee
23	29	W	6	Secondary	Employee
24	26	W	1	Secondary	Employee
25	40	W	13	Secondary	Employee

^**a**^W woman

^**b**^M man

**Table 2:** Demographic data of interviewed parents.

Parent					Child							
Participant number	Age	Gender	Education level^d^	Adherence to Dutch Guidelines for Healthy Physical Activity	Age	Gender	Diagnosis	Walking pattern	FMS^c^			Adherence to Dutch Guidelines for Healthy Physical Activity
1	31	W^a^	High	No	5	F	None	Normal	6	6	6	Yes
2	36	W	High	No	4	F	Unknown	Atypical, wheelchair	C	2	1	No
3	35	W	Middle	Yes	4	M	Brain damage, hypermobility	Atypical, wheelchair	5	1	1	Yes
4	45	W	High	No	7	F	Syndromal disorder	Normal	6	6	6	Yes
5	36	M^b^	Low	No	13	M	Obesity	Normal	6	6	6	No
6	42	W	High	No	11	F	Syndromal disorder	Atypical, wheelchair	5	2	1	No
7	49	W	High	No	10	F	Spina Bifida	Normal	6	6	6	Yes
8	29	W	Middle	Yes	3	M	Cerebral Palsy	Atypical	6	5	5	Yes
9	25	W	Middle	No	9	F	None	Normal	6	6	6	Yes
10	42	W	Low	No	15	F	None	Normal	6	6	6	Yes
11	49	M	High	Yes	4	F	Arthrogryposis multiplex congenita	Atypical, wheelchair	C	1	1	No
12	46	W	High	No	10	M	Spina Bifida	Wheelchair	1	1	1	No

^**a**^W woman

^**b**^M man

^c^FMS: Functional Mobility Scale [29]. C: crawling; 1: uses wheelchair; 2: uses a walker or frame; 3: uses crutches; 4: uses sticks; 5: independent on level surfaces; 6: independent on all surfaces.

^d^Educational level in the Netherlands. Definition of Low: first three years of senior general secondary education and pre-university secondary education; the various pathways of prevocational secondary education including lower secondary vocational training and assistant’s training [36]. Definition of Middle: upper secondary education, basic vocational training, vocational training, and middle management and specialist education [36]. Definition of High: associate degree programs, higher education, Bachelor programs; 4-year education at universities of applied sciences; Master degree programs at universities of applied sciences and at research universities; and doctoral degree programs at research universities [36].

**Table 3:** Demographic data of interviewed children.

Participant number	Age	Gender	Diagnosis	Walking pattern	FMS^c^			Adherence to Dutch Guidelines for Healthy Physical Activity	Education (regular/special)
1	17	G^a^	Visual impairment	Normal	6	5	5	No	Mainstream
2	10	G	None	Normal	6	6	6	Yes	Mainstream
3	8	G	None	Normal	6	6	6	No	Mainstream
4	12	B^b^	Unknown developmental retardation	Wheelchair	1	1	1	No	Special
5	17	B	Cerebral Palsy	Wheelchair	1	1	1	No	Mainstream
6	9	G	Spina Bifidia	Normal	6	6	6	No	Mainstream
7	9	G	Brain disorder	Normal	6	6	6	Yes	Mainstream
8	12	G	None	Normal	6	6	6	Yes	Mainstream
9	14	G	Nephrological failure	Normal	6	6	6	No	Mainstream
10	12	G	Cerebral Palsy	Atypical	5	5	5	No	Special
11	6	B	None	Normal	6	6	6	No	Mainstream
12	16	B	None	Normal	6	6	6	Yes	Mainstream

^**a**^G girl

^**b**^B boy

^c^FMS Functional Mobility Scale [29]. 1: uses wheelchair; 2: uses a walker or frame; 3: uses crutches; 4: uses sticks; 5: independent on level surfaces; 6: independent on all surfaces.

### Procedure

We developed a topic guide based on Fleuren et al.’s ‘Determinants of innovation within health care organizations’ [37] as well as on the expert knowledge of the research team [38, 39]. We asked participants about their opinion regarding the current design of the AM-p and its development, expectations regarding necessary instruction material, output and use in daily living (data in S1 Appendix). The topic guide was short, as we wanted to give interviewees the freedom to express their own thoughts and needs related to the AM-p. We collected individual semi-structured interviews with two researchers, conducted by the first author and four trained PPT Master’s students of the University of Applied Sciences in Utrecht to enhance reflexivity [35]. Each researcher involved in data collection pilot-tested the topic guide with one or two interviews.

Due to COVID-19 restrictions, we conducted interviews with PPTs and parents digitally via a video-call (Microsoft Teams) recorded with FreeCam 8 for Windows (iSpring Solutions Inc., Alexandria, United States). Before the interviews, we displayed a photograph of the AM-p on the screen to familiarize PPTs and parents with its current design (S1 Fig). We interviewed children face-to-face to reduce the abstractness of the words ‘activity monitor’ and provided them with the possibility to see, feel and wear the AM-p. We encouraged all children and adolescents to draw pictures to complement and facilitate verbal expressions about the design and output of the AM-p [40]. During interviews, children and adolescents were free to choose having their parents present or not.

### Data management and analysis

We recruited participants until data saturation within our sample was reached, defined as when no new themes derived from the latest two interviews [41]. We recorded all interviews with audio equipment and saved it in secured workspaces. We used Amberscript (Amberscript B.V., 2021, San Francisco & Amsterdam) to transcribe all audio recordings. We anonymized each transcript and controlled and corrected it, using a standardized protocol to ensure verbatim transcription. An independent researcher checked the data to ensure correctness of transcription and anonymization. We analyzed the data using ATLAS.ti 9 for Windows (Scientific Software Development GmbH, Berlin, Germany), as well as manual strategies, such as mind-mapping.

We analyzed data with an inductive approach, following the six steps of Braun and Clarke’s reflexive thematic analysis [29]. First, we read the transcripts several times to familiarize ourselves with the data. Second, we independently identified fragments and generated initial codes with two researchers. Third, we grouped codes into potential themes with three researchers, ensuring investigator triangulation [35], consulting a fourth if consensus could not be reached. Fourth, we conducted detailed reviewing of the themes in relation to individuals and to the entire data set. Fifth, we generated clear definitions and names for each theme. We held several peer debriefing sessions with the whole research team to discuss and define themes to increase confirmability [35]. We used the Fleuren’s determinants as framework to cluster the themes. Sixth, we chose quotes related to the different themes. For confirmability, we provided a summary of the main results to a group of PPTs and parents, and no new themes emerged from the subsequent discussion [35]. Due to COVID-19 restrictions, group meetings were not allowed. Thus, we did not verify results for children and adolescents in group sessions.

## Results

We interviewed 25 PPTs, 12 parents, 12 children and adolescents. On average, PPTs were 38 (SD 10) years of age, parents 39 (SD 8), and children and adolescents 12 (SD 3). Interviews lasted on average 47 (SD 15) minutes.

We identified nine relevant themes, categorized into four of the determinant groups of Fleuren et al: 1) determinants related to the innovation, 2) determinants related to the end-user, 3) determinants related to the organization, 4) determinants related to the socio-political context. Themes that could not be categorized are summarized in “other”. In the text below, quotes are given in quotation marks.

### 1. Determinants related to the innovation

#### 1.1 Development of information materials

Overall, end-users required information material with instructions and explanations for successful use of the AM-p in daily living (e.g., digital information material, booklets/flyers). For primary-school-aged children or children and adolescents with intellectual disabilities, colorful lay-outs with simple instructions and pictures were preferred. To facilitate adequate information flow and ensure reliable measurements, parents and PPTs needed information material that could be used for third parties. End-users said that information should be short, clear and relevant for practical daily use of the AM-p.

#### 1.2 Application: Output visualization and ease of use

To extract relevant information from an assessment, an application with clear and easy-to-read output was essential. PPTs pointed out that the application should be self-explanatory or at least easy to use, especially for colleagues with little affinity for technology. The output of a measurement should be visualized by graphs or charts, and easy to understand for all end-users. Primary-school-aged children preferred insight into their activities with pictures or cartoons. PPTs wanted to be able to differentiate between various movement categories, depending on the goal of a measurement (e.g. global difference between activity and inactivity, or basic activities such as lying, sitting, walking, running, biking, and propelling a wheelchair). Also, PPTs and some parents required insight into the amount of physical activity per hour, day or week. Presenting too many details could be overwhelming and difficult to understand for some end-users.

*PPT#4*: *“I think*, *often we expect too much from parents and children*. *They actually don’t want to have too much information*. *They just want to know*: *am I active enough or not*?*”*

Parents of children with a developmental disability seemed interested in more detailed information about an assessment than parents of children without a developmental disability.

#### 1.3 Design

*1*.*3*.*1 Outer appearance of the AM-p*. Opinions were divided on the current design of the AM-p. Most adolescents and parents of children who have or are familiar with a physical disability thought that the current design was fine: they were already used to being different and so the AM-p would have no huge influence on their overall appearance.

*Parent#3*: *“He attends school in his wheelchair [*…*] he is already different from other children who are going to school*. *He is already special*, *so it will not matter if he wears something additional* [i.e. activity monitor].*”*

Other end-users were more critical of the current design: the prototype was bulky, child-unfriendly, and looked boring. They assumed that a smaller and lighter design would have a positive influence on its acceptability to children and adolescents.

*PPT#4*: *“[…] if you are talking about the target group of very young children at the age of four years–well*, *maybe they like it [AM-p] when it’s colorful and fun to look at*. *But when you’re talking about a teenager*, *he or she wants something that is as least noticeable as possible*. *Therefore*, *when you have a rather large monitor on your ankle*, *it almost looks as if you’re a juvenile delinquent*. *So*, *it would be clever to have something very small that you can’t see* [for the teenagers] *while it could be something ‘cool’ to wear for a younger child*.*”*

All end-users agreed that for primary-school-aged children the design of the AM-p should be colorful, with a choice of different straps in multiple colors or designs.

*1*.*3*.*2 Attachment to the body*. The development of a child-friendly strap and casing to attach the AM-p to the body was important. End-users agreed that young children should not be able to open the strap. Adolescents, on the other hand, should be able to attach the AM-p independently and take responsibility for wearing it. Opening and closing the strap should be possible for end-users with different abilities, e.g. with limitations in hand use, or visual impairments. The material of the strap and casing should be durable, hygienic, resistant to cleaning fluids, comfortable, not irritating to the skin nor making it sweaty.

*1*.*3*.*3*. *Wearing comfort*. A good fit and comfort when wearing the AM-p were very important to all end-users. Some PPTs and parents believed a child’s performance of activities might be affected negatively by an uncomfortable AM-p.

Children and adolescents did not encounter a problem to regularly charge the AM-p’s battery, comparing it to the routine of charging a mobile phone. Some parents and children were worried about forgetting to charge it, seeing it as an additional, though manageable, burden in their daily life.

*Child#12*: *“I think that I could manage* [to charge it]. *Well*, *it also depends on how seriously you take the assessment*. *If I don’t take it seriously*, *then I would probably forget* [to charge the AM-p].*”*

### 2. Determinants related to the end-user

#### 2.1. Relevance & acceptance

*2*.*1*.*1*. *PPTs*. Overall, PPTs thought that using the AM-p could be an important assessment for individual goal-setting and therefore relevant for use with different patient groups. A PPT thought that an AM-p assessment could be useful, but might result in too much additional work for children with developmental disabilities and their parents. Another PPT saw surplus value from working with the same AM with facilitation of collaboration among colleagues in different work settings:

*PPT#22*: *“A while ago*, *I got a report from a PPT working in primary care; she took many tests over a few months*. *That was very insightful* [for me] *because I see the child just for a very short moment* [at the specialist center]. *Back then*, *I had information about the development over time*. *If a PPT in primary care could capture the longitudinal output of the AM-p in a report… it can provide us with information about the progression of an illness*.*”*

There was also doubt among some PPTs about the added value of the AM-p in clinical practice:

*PPT#18*: *“I think it is important to have insight into the activity level of a child during the day*. *[…] But if it has to be with such an AM-p*, *[…] I have my doubts about it*, *because I don’t see the additional value of that box* [AM-p] *for a PPT*.*”*

To be able to use the AM-p successfully in daily clinical practice, many PPTs expressed a need for training in the technological aspects of the hard- and software, psychometric properties, interpreting results and communication skills during the clinical reasoning process.

Overall, PPTs thought that colleagues would be interested in using the AM-p and were willing to strive for more objective assessment results and a higher level of health care service. They also assumed that their colleagues’ enthusiasm for the AM-p would depend on PPTs’ age, their affinity with technology, the sort of patients they saw, and their willingness to step out of their comfort zone.

*2*.*1*.*2 Children*, *adolescents*, *and parents*. Parents of children with a developmental disability saw more benefit in monitoring their child’s PA than did parents of children without developmental disabilities. Nevertheless, the relevance of working with the AM-p had to be evaluated and weighed with regard to personal circumstances.

*Parent#6*: *“[*…*] sometimes*, *I am asking myself*: *how much movement is she actually getting at home*, *isn’t she sitting too much in her wheelchair and is that*… *let’s say*, *okay for her*?*”*

It was important that parents and children understood why the PPT was proposing the AM-p as an assessment tool. When parents and children were convinced that an AM-p assessment would provide valuable insights for the child, they seemed willing to use it.

Ideas about its potential acceptance by peers varied. Parents of children with a developmental disability thought their child’s peers would react positively to, or at least be interested in, the AM-p as they were already aware that the child was ‘different’. Adolescents with a developmental disability expect the same:

*Child#10*: *“It doesn’t matter; for example*, *my braces*: *if they* [peers] *ask what that is*, *I explain it to them and then they know*. *And then they just know and it’s done*.*”*

Parents of children without a physical disability or with an invisible disability had more doubts about acceptance of peers. One child and parent were afraid that the AM-p would draw attention to already insecure children who were easily victims of bullying. Despite the positive personal attitude towards the AM-p, one adolescent was afraid that wearing the AM-p on the ankle might raise negative associations among other people.

#### 2.2 Shared decision-making

All end-users emphasized the critical role of the PPT during the introduction of the AM-p. It had to be transparent and clear why the PPT wanted to use the AM-p as an assessment tool. Parents and PPTs were convinced that the interaction between all end-users was an important start for a successful assessment. A mother thought that she might easily feel offended when a PPT proposed to use an AM-p to monitor her child’s PA. Transparent communication was essential for shared decision-making, mutual understanding, and a non-judgmental atmosphere.

*Parent#6*: *“[*…*] especially*, *if you develop a roadmap for her*, *like*: *we do it like this*. *Then*, *she is prepared to* [wear] *it and has involvement*. *Yes*, *especially that she’s involved […] and that she has a grip on it and is a bit responsible for it* [assessment], *then you can encourage her for sure* [to wear the AM-p].*”*

#### 2.3 Compatibility in daily living

According to all end-users, the AM-p should ideally be compatible with diverse activities a child performed in daily living: playing indoors and outdoors, climbing, running, swimming, jumping, riding a (hand)bike, propelling a wheelchair, or playing soccer and hockey (sports). Parents, children and adolescents worried that the AM-p might get damaged when wearing it for an assessment.

*Child#8*: *“That you can do just everything you usually do in your life without that thing* [AM-p] *ruining it*.*”*

To facilitate compatibility, the AM-p should ideally be protected against sand, water, dirt, and must be able to withstand collisions.

*Parent#7*: *“Yes*, *it should withstand a collision […] Yes*, [children] *climb in trees*, *run through puddles and play soccer*. *And if you’re playing soccer in a tackle and somebody wants to get the ball*, *you just get a kick to your ankles*.*”*

### 3. Determinants related to the organization

#### 3.1 Finances

The financial value of the prototype to the PPTs varied, depending on the additional value to the working process, ease of use, options for displaying the output, and the financial capacity PPTs had in their working environment. Depending on the surplus value of the end-product, PPTs were willing to invest between 25 and 500 euro per AM-p. A few parents and PPTs were concerned about the financial consequences if an AM-p got damaged or lost during an assessment. PPTs were also worried about the huge number of AM-ps they might need to buy at one time in order to assess the baseline level of a therapy group.

#### 3.2 Time

The time factor was of major importance, especially for PPTs working in primary care. If using the AM-p reduced the workload, PPTs were willing to invest time and energy in becoming proficient in using the AM-p and concomitant tools, such as the web application and information material.

*PPT#1*: *“For me*, *it is a waste of time if it takes an hour of my time to organize*, *interpret and record the output in a report*. *Then I would use it less because I just won’t get it done […]*. *I just don’t have the time for it*, *I think*.*”*

### 4. Determinants related to the socio-political context

#### 4.1 Legislation and regulations

Parents and PPTs expected the developer of the AM-p to fulfill all legislative requirements and regulations needed for its safe use, e.g. General Data Protection Regulation. Parents expected the PPT to be able to answer their questions about privacy.

### 5. Other themes

For the further development of the AM-p, PPTs, parents and adolescents had multiple ideas and interests: they required, e.g., insight into the intensity of activities, and wanted reference values for assessments, as well as 24/7 monitoring to get better insight into activity and sleep. There was also interest in using the AM-p not only as assessment tool, but also as an intervention tool with real-time feedback.

## Discussion

The purpose of this study was to explore needs and requirements for the AM-p in everyday PPT clinical practice among children, adolescents, parents and PPTs. We found nine themes within four groups of determinants, as follows: Determinants related to the innovation: 1) development of information materials; 2) application: output visualization and ease of use; 3) design; determinants related to the end-user: 4) relevance and acceptance; 5) shared decision-making; 6) compatibility in daily living; determinants related to the organization: 7) finances; 8) time; determinants related to the socio-political context: 9) legislation and regulations.

We encouraged participants to think in an unlimited way about their needs and requirements for the AM-p. In a next step, input from this study will be used to decide with relevant stakeholders, including developers and budget holders, which needs and requirements can be fulfilled. Therefore, researchers, PPTs, parents, children, and adolescents should be enabled to elaborate mutual needs together [42, 43]. Based on feasibility and available resources, researchers and developers will decide which of the needs should be incorporated within the innovation.

End-users mentioned an added value of the AM-p as an assessment tool in PPT practice. To facilitate use of the AM-p, it is important to develop such concomitant tools as information material, an easy-to-use web application and child-friendly straps for attaching the AM-p to the body. The tools should be customized to different target groups. The age of the children involved seems important to consider during further development of the AM-p: primary-school-aged children and their parents preferred colorful and fun appearances, whereas adolescents preferred a simple, unobtrusive design. Furthermore, end-users with a developmental disability wanted fewer barriers to wearing the AM in daily living than end-users with typical motor development. This implies that a diverse group of end-users should be involved in the further development of the AM-p [44, 45]. Ideally, end-users would be equal partners of the development team, together with co-designers and other researchers, and would be involved in the developmental starting phase of the concomitant tools. For this reason, input about the specific context of end-users’ personal needs and requirements would be warranted [42, 46, 47].

End-users pointed out that appropriate communication skills of PPTs would be essential when introducing the AM-p. All end-users emphasized the importance of providing children, adolescents, and parents with appropriate information about the surplus value of using the AM-p as an assessment tool. Shared decision-making appeared important when integrating the AM-p into patient care. This would enhance not only the benefit to the health care provider but also the authority of the family and the autonomy and well-being of the child [48]. Our observations indicate that PPTs could involve all end-users in shared decision-making with transparent communication, which is important for a successful therapeutic relationship. This is recently supported by Boland et al., who stated that information provided by health care professionals should contain presentation of options, their associated risks and benefits, and research evidence. The information provided should be appropriately tailored to the child’s developmental needs and to the child and parents’ level of health literacy [49].

We chose Fleuren et al.’s ‘determinants of innovation within health care organizations’ as a base for our topic guide, organizing our identified themes into four of their groups of determinants [37]. In this early developmental stage of the AM-p, most important themes for all end-users are linked to ‘determinants related to an innovation’ and ‘determinants related to the end-user’. ‘Determinants related to the organization’, such as time and finances, were mainly related to the limited time PPTs are able to invest when working with the AM-p. PPTs have to invest time to become skilled with the use of the AM-p, understand and use a web application, and be able to analyze, interpret and communicate about results. In the further development, time-efficiency when using the AM-p should be addressed thoroughly. As the AM-p is not yet ready for use, the financial value varies enormously among PPTs, depending on the amount of devices they have to buy and financial background. Training is essential for most PPTs, but reported disadvantages of proper training were mainly related to the time this would take, with, as a result, loss of paid working time. Literature about implementation of technology in health care showed proper training of health care professionals to be essential: training PPTs would ensure adequate use of an AM, proper instruction of patients, and a feeling of competence in using an AM [11, 50]. A blended learning approach might contribute to the individual training needs of PPTs, e.g. flexible self-study, face-to-face hands-on lessons, with possibilities for asking questions (for example through an online consultation by video link). Blended learning enables learners to work flexible with regard to sequence, pace, and time of their learning [51, 52].

This study has several strengths and limitations. We managed to include participants with diverse backgrounds for age, presence of developmental disability, adherence to the Dutch Guidelines for Healthy PA, education level, work setting, and work experience. Also, we were able to interview parents of children younger than five years, particularly interesting and important because lifestyle habits established early in life provide an opportunity to shape lifestyle habits through childhood, adolescence and into adulthood [53]. However, our participants may have had a pre-existing interest in physical activity and activity monitoring which could have influenced the results. Furthermore, our sample did not have diverse cultural backgrounds which could have influenced our results. Within the sample of children, we interviewed mainly adolescents with developmental disabilities. A broader variety of younger children (e.g., 6 to 10 years old) with atypical walking pattern, using a wheelchair in daily living, or attending special education could have enriched the obtained information. Inclusion within this group of children was difficult due to COVID-19 pandemic. For future research, we recommend to include a more varied sample of children than we were able to obtain.

Due to the COVID-19 pandemic, we conducted online interviews with PPTs and parents. In online interviews, responses may have been shorter and may have provided less contextual information [54]. However, given the data saturation within our sample, we assume conducting the interviews online did not greatly influence the answers of our participants [55].

The development of a generic AM as an assessment tool to measure PA in children and adolescents in PPT clinical practice is complex. We encourage researchers to include participants with different cultural backgrounds. This may lead to broader insights and could enhance a greater transferability to the culturally enriched environment a PPT works in. Potential cultural and language barriers could be identified early and incorporated in the developmental process, e.g. inclusiveness of information material. For the same reason, we recommend to include a broader variety of children with developmental disabilities (atypical movement patterns, attending special needs schools, even distribution of children and adolescents). If possible, live interviews should be held. We tried to familiarize all participants with a picture of the AM-p to reduce abstractness and introduce the device. We obtained the idea that children (and present parents) who saw, felt and tested the AM-p during an interview knew very well what they were talking about. We aim to develop a generic AM assessment tool for PPTs. Although technicians can develop software that is capable of handling the variety of end-users requirements, this study showed that the concomitant tools and the design of the AM-p should be tailored to the different needs of children, adolescents, and their parents.

### Actionable insights

- Time is of major importance for PPTs: added up, they have to invest much time to become skilled with the use of the AM-p. Understanding a web application, analyzing results and interpreting them has to be easy and time efficient. Therefore, the web application must be very easy to use for all PPTs. Results of an assessment have to be presented to a child and parents preferably within one contact moment. The output must be visualized and transparent, thus inclusive for children, adolescents and people with low literacy or language barriers.
- Training is essential for most PPTs but has to be as efficient and short as possible.
- The wearing comfort of the AM-p and its compatibility in daily living is essential, a child or adolescent should not be restricted in his or her daily activities when wearing the device.
- Information material with instructions should be short, clear and relevant for practical daily use of the AM-p.
- Shared decision-making and mutual understanding can be enhanced by transparent communication of PPTs during the introduction of the AM-p.

## Conclusion

The AM-p has potential in PPT daily clinical practice for all end-users. Children, adolescents, parents and professionals have similar basic needs, with individual requirements for further development. Concomitant tools, such as a suitable strap, information material and an easy-to-use application to read and interpret results, are needed. Further development of the design should be child-friendly and compatible with daily living. The relevance of using the AM-p needs to be explained by knowledgeable PPTs as part of the shared decision-making process. This can support acceptance of the use of the AM-p. We recommend involving PPTs, parents, children, and adolescents in all developmental stages of the AM-p and concomitant tools to enable further development to be customized as much as possible.

## Supporting information

S1 Appendix Topic guide. (DOCX)

S1 Fig Familiarization AM-p interview. Red circles highlight the AM-p.
(TIF)
