# Supplementary material for: Exploring needs and requirements for a prototype device measuring physical activity in pediatric physical therapy: A qualitative study
Source: PLoS One. 2024 Jun 25;19(6):e0305968. doi: 10.1371/journal.pone.0305968 (PMC11198827; doi:10.1371/journal.pone.0305968)
Supplement: S1 Appendix — (DOCX) [file pone.0305968.s001.docx]

Appendix S1

Topic list pediatric physical therapist

*Experience, current assessments of physical activity*

How are you assessing the physical activity of children/adolescents at the moment?

- Clinimetrics
- Patient groups
- Experience: what works, what is important, pros and cons

*Expectations/wishes related to activity monitor prototype*

- Value for therapists
- Difference between primary, secondary and tertiary care
- What do you want to be able to measure with the prototype?
- How do you want the output to be?
- Which details do you need?
- Dream: what does the perfect activity monitor look like, what is it able to do?

*Ease of use*

- Time investment in training of pediatric physical therapists -> what works best?
- Time investment using activity monitor prototype: introduction, analysis of results
- Feedback with patient on results -> what do you need?
- Design of activity monitor prototype

*Reactions of parents/child/adolescent*

- Satisfaction/acceptance of activity monitor prototype (why/why not?)
- Expectations when using activity monitor prototype

*Use in daily clinical practice, future*

- Use of activity monitor prototype in the future (therapist him/herself and/or colleagues)

*Finances*

- Financial value
- Responsibility

*Activity monitor prototype*

- Ideas about it
- Points of improvement
- Requirements
