# Supplementary figures and images for: Exploring needs and requirements for a prototype device measuring physical activity in pediatric physical therapy: A qualitative study
Source: PLoS One. 2024 Jun 25;19(6):e0305968. doi: 10.1371/journal.pone.0305968 (PMC11198827; doi:10.1371/journal.pone.0305968)

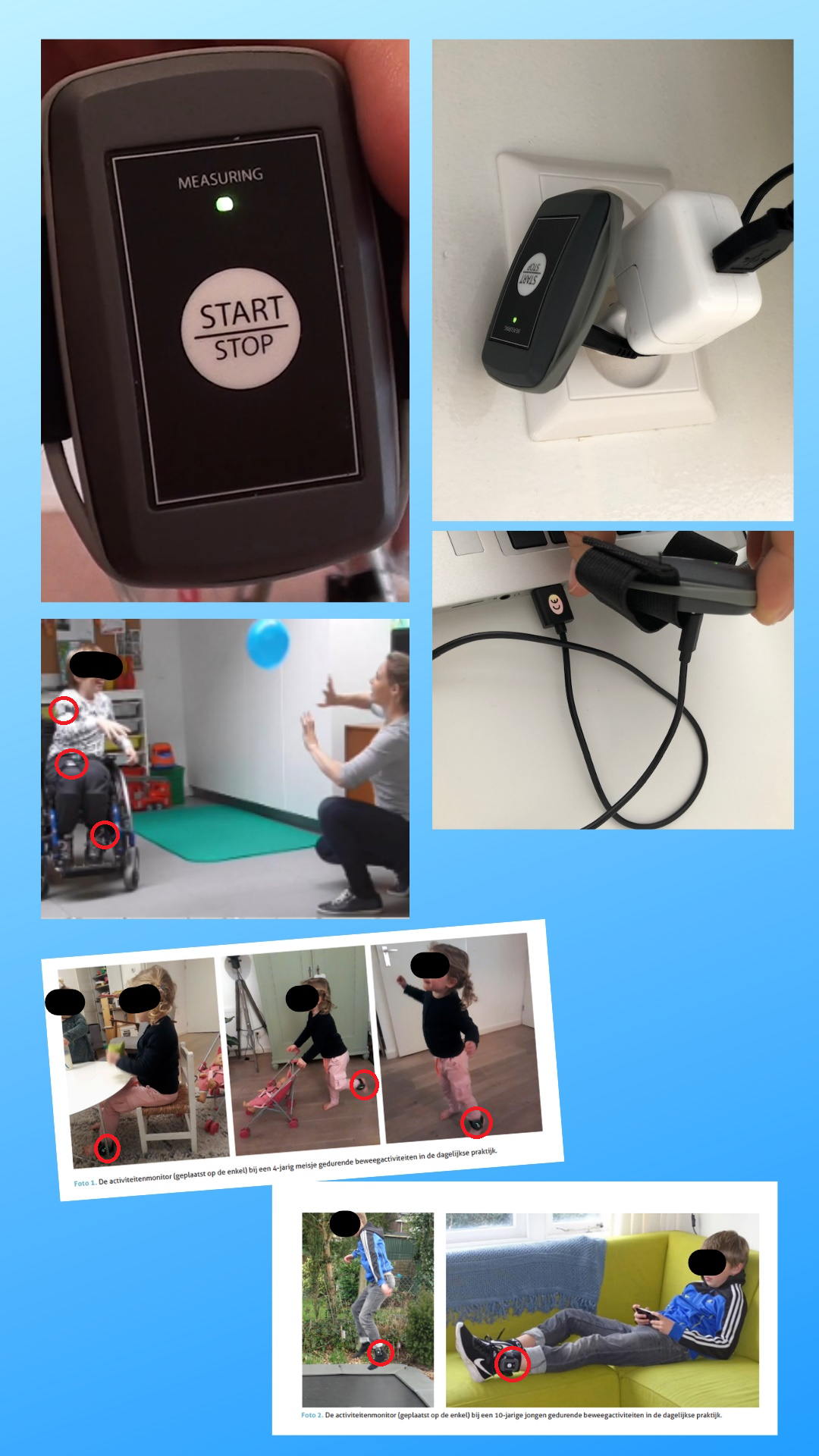

Supplement: S1 Fig — Red circles highlight the AM-p. (TIF) [file pone.0305968.s002.tif]
